# Supplementary figures and images for: Embryo and larval biology of the deep-sea octocoral Dentomuricea aff. meteor under different temperature regimes
Source: PeerJ. 2021 Aug 2;9:e11604. doi: 10.7717/peerj.11604 (PMC8340903; doi:10.7717/peerj.11604)

**A**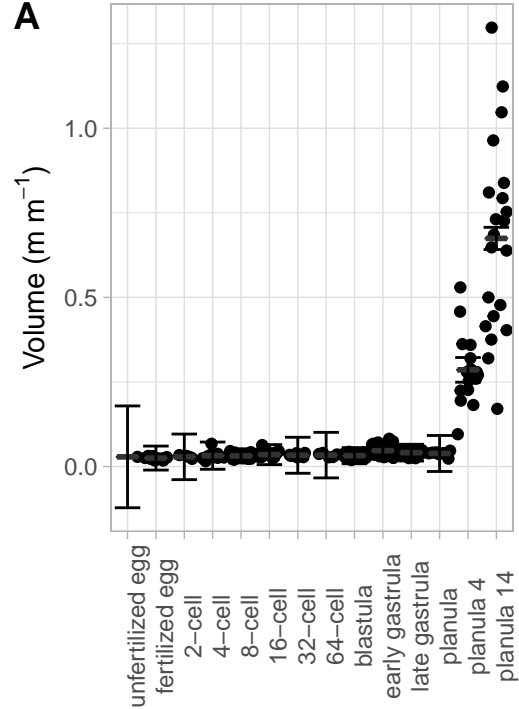**B**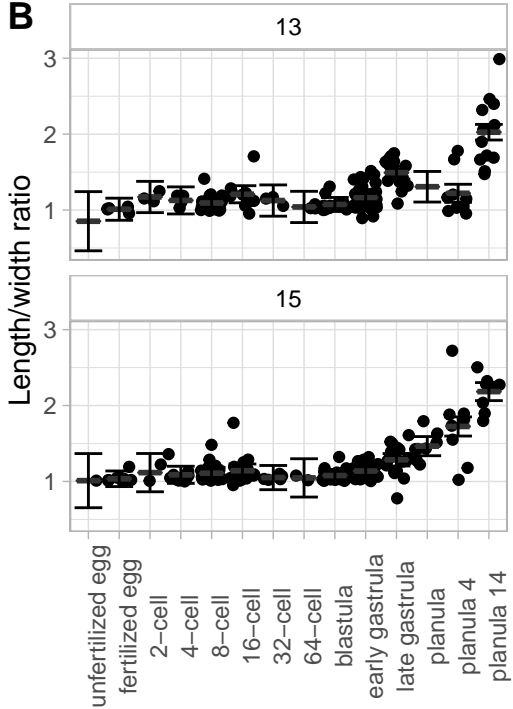

Supplement: Supplemental Information 3 — Lines represent model fit and 95% confidence intervals, while points represent model partial residuals. [file peerj-09-11604-s003.pdf]

13°C

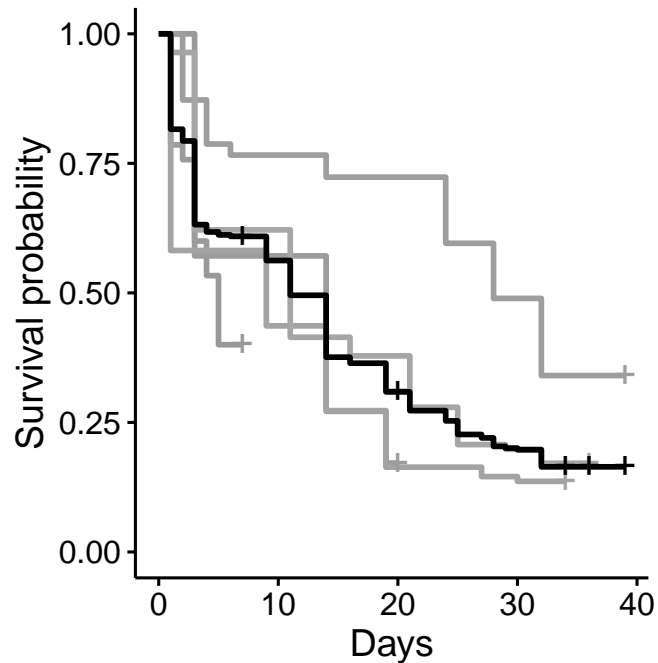

15°C

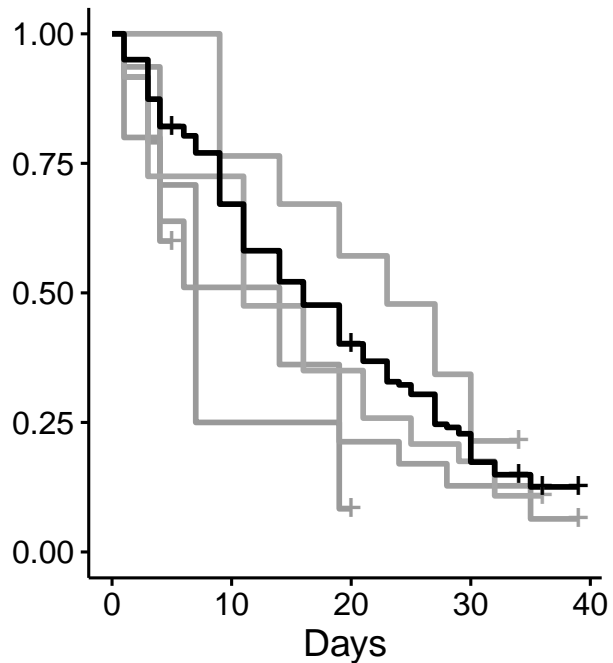

Supplement: Supplemental Information 4 — Grey lines represent estimates for separate batches while black lines represent estimates for all data pooled together. [file peerj-09-11604-s004.pdf]

13°C

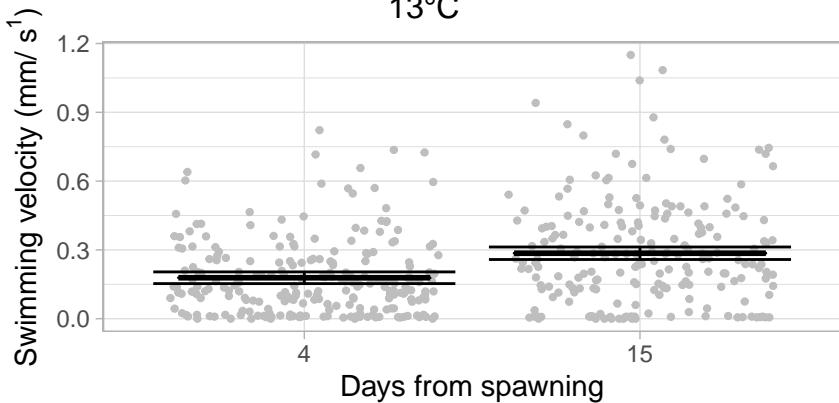

15°C

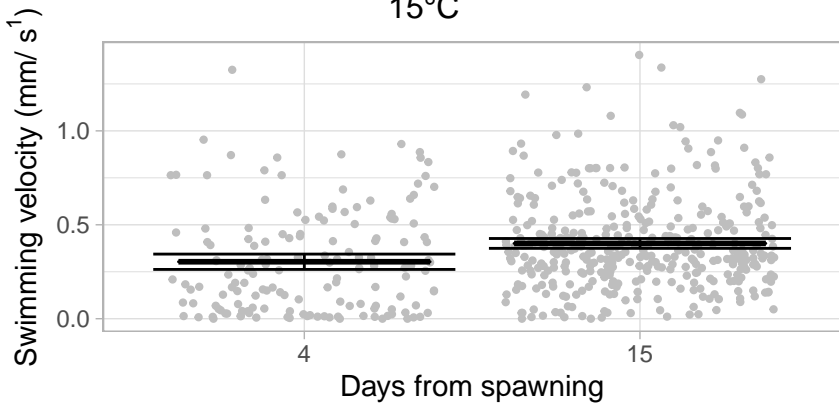

Supplement: Supplemental Information 5 — Lines represent model fit and 95% confidence intervals, while points represent model partial residuals. [file peerj-09-11604-s005.pdf]

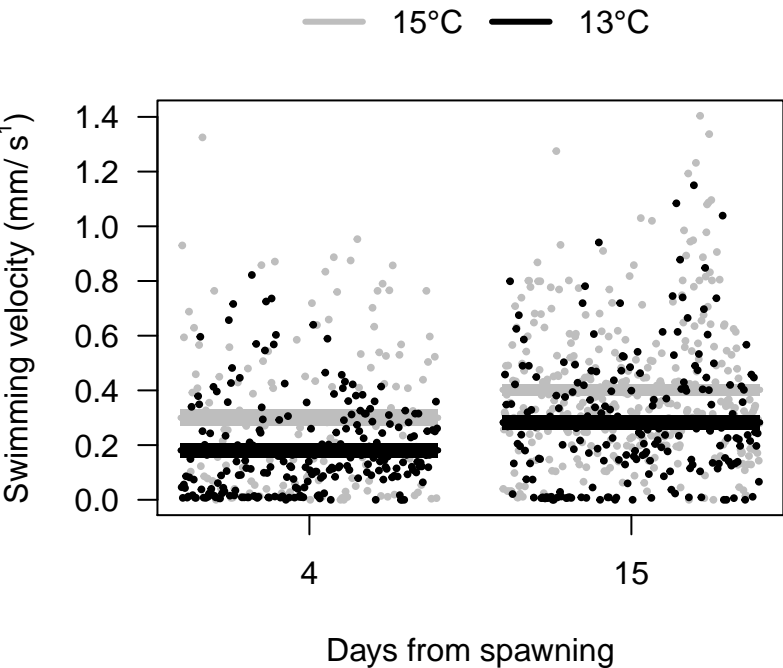

Supplement: Supplemental Information 6 — Lines represent model fit and 95% confidence intervals, while points represent model partial residuals. [file peerj-09-11604-s006.pdf]

Deformed

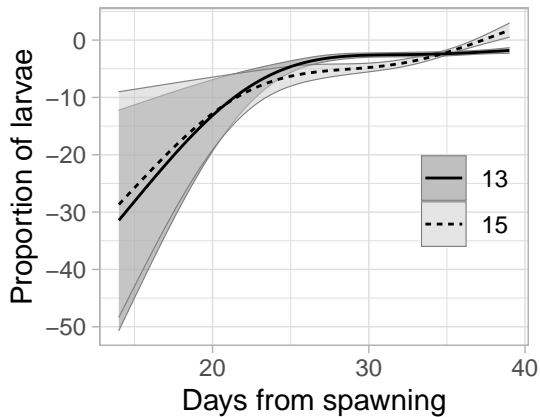

Metamorphosed

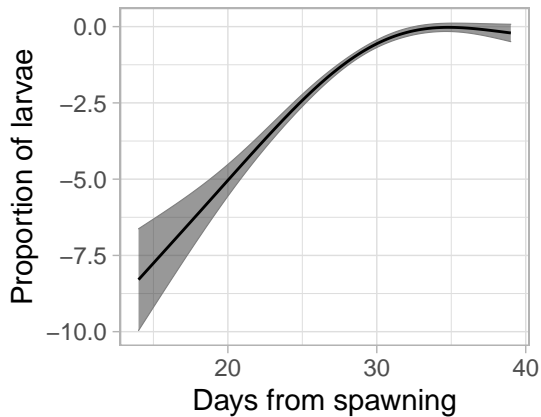

Planula

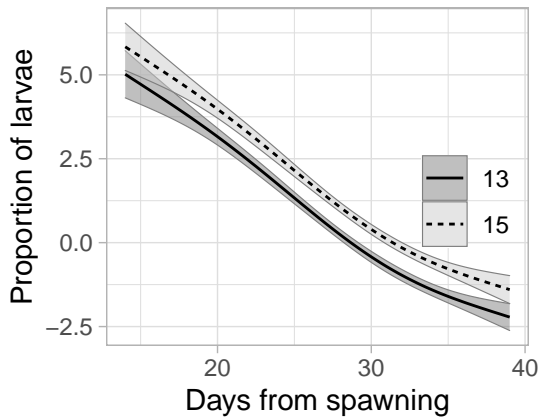

Settled

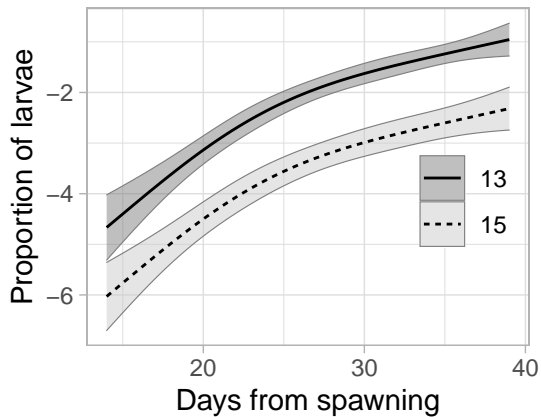

Supplement: Supplemental Information 7 — Lines represent model fit and 95% confidence intervals. [file peerj-09-11604-s007.pdf]
